# The Role of Oxytocin and Cortisol as Biomarkers of Efficacy in Animal-Assisted Interventions for Patients With Major Depressive Disorder: A Narrative Review

**DOI:** 10.7759/cureus.103217

**Published:** 2026-02-08

**Authors:** Barbara Balajewicz, Sara Szukalska, Marta Karczewska, Angelika Samborska, Kamil Wróblewski, Lukasz Siwek, Paulina Wróblewska, Karolina Lichwala

**Affiliations:** 1 General Medicine, Medical University of Silesia, Katowice, POL; 2 Orthopaedics and Traumatology, Dr. Tytus Chałubiński Specialist Hospital in Radom, Radom, POL; 3 General Medicine, Poznan University of Medical Sciences, Poznan, POL; 4 General Medicine, Medical University of Silesia, Zabrze, POL; 5 General Medicine, University Clinical Hospital in Poznan, Poznan University of Medical Sciences, Poznan, POL

**Keywords:** animal-assisted therapy, biomarkers, cortisol, major depressive disorder, oxytocin, psychoneuroendocrinology

## Abstract

Major depressive disorder (MDD) is a leading cause of disability worldwide, often characterized by dysregulation of the hypothalamic-pituitary-adrenal (HPA) axis and impaired social affiliation. Despite standard pharmacological treatments, a significant proportion of patients continue to struggle with treatment-resistant depression, necessitating the exploration of effective adjunctive strategies such as animal-assisted interventions (AAIs).

The primary objective of this narrative review is to synthesize current neurobiological evidence regarding the efficacy of AAIs in MDD populations. Specifically, we aim to evaluate the modulation of cortisol and oxytocin as primary biomarkers and to delineate the role of C-tactile (CT) afferent pathways in mediating these physiological shifts.

A comprehensive literature search was conducted across PubMed, Scopus, and Web of Science (2000-2026), focusing on quantitative studies and meta-analyses investigating neuroendocrine responses to AAIs in depressive or high-stress populations.

The evidence indicates that even brief (10-20 minute) interactions with animals can lead to a significant reduction in salivary cortisol levels, suggesting rapid HPA axis stabilization. Furthermore, oxytocin secretion, stimulated by visual and tactile contact, enhances emotional regulation. This process is further mediated by the activation of CT-tactile afferents during petting, which project to the insular cortex to promote anxiolytic effects. Comparative analysis suggests that while canine-assisted interventions yield acute stress reduction, equine-assisted therapies may influence long-term diurnal cortisol rhythms.

AAIs offer a measurable, biologically grounded supportive treatment for MDD. While promising, the field requires standardized protocols and large-scale longitudinal studies to establish evidence-based guidelines for clinical psychiatric practice.

## Introduction and background

Major depressive disorder (MDD) is a complex psychiatric condition characterized by core symptoms including persistent low mood, anhedonia, and fatigue, as defined by the DSM-5 criteria. Diagnosis requires the presence of at least five symptoms during the same two-week period, causing significant clinical distress. MDD represents a formidable challenge to global public health, affecting approximately 280 million people worldwide [1]. A critical challenge in clinical practice is treatment-resistant depression (TRD), typically defined as a failure to achieve symptomatic remission despite at least two trials of antidepressant therapy of adequate dose and duration. Despite the availability of diverse pharmacological modalities, an estimated 30% of patients fail to respond adequately, falling into the category of TRD [2]. While meta-analyses confirm the efficacy of various antidepressant drugs [3], many clinical outcomes remain suboptimal, necessitating the exploration of effective adjunctive strategies [4].

**Figure 1:**
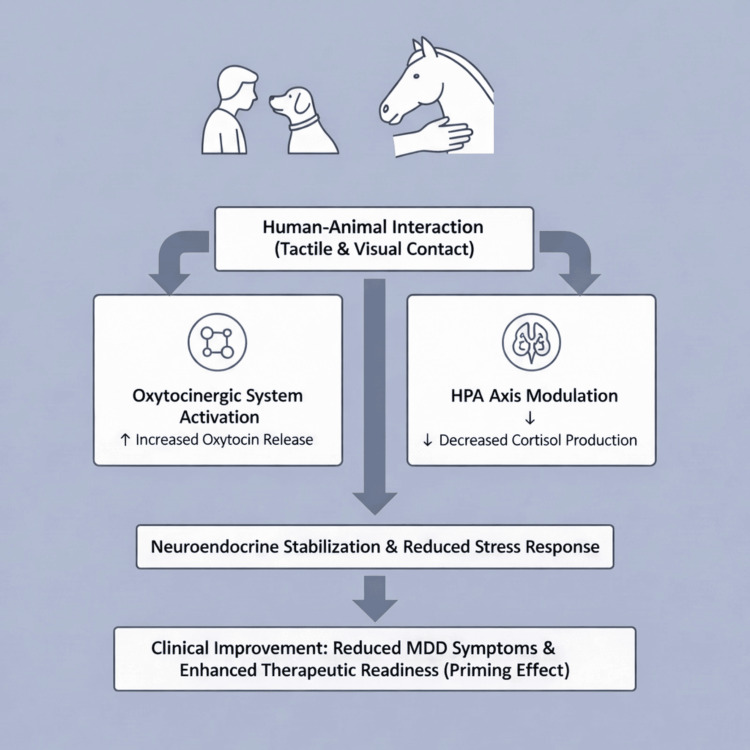
Neurobiological Mechanisms of Animal-Assisted Interventions (AAIs) in the Treatment of Major Depressive Disorder (MDD). Figure created by the author using the Google Gemini AI platform. HPA: Hypothalamic-pituitary-adrenal

Beyond conventional therapies, animal-assisted interventions (AAIs), an umbrella term for structured modalities such as animal-assisted therapy (AAT), which is goal-directed and professional-led, and animal-assisted activities, which focus on motivational visitation, have gained recognition as a promising supportive approach [5]. While the psychological benefits of human-animal interaction are well-documented, its therapeutic potential in MDD is primarily linked to the stimulation of the oxytocinergic system and the modulation of the hypothalamic-pituitary-adrenal (HPA) axis [6,7]. The release of oxytocin acts as a natural modulator, providing a physiological counter to the chronic hypercortisolemia and stress response frequently observed in MDD populations [8,9]. Previous studies have shown that even brief interactions can influence salivary cortisol secretion and social engagement markers [10,11]. However, the neurobiological pathways of human-animal interaction and the role of early life stress in HPA axis dysregulation remain critical areas of investigation in psychiatric populations (Figure 1) [12].

## Review

Methodology

To ensure transparency and scientific rigor, a literature search was conducted across PubMed, Scopus, and Web of Science for articles published between January 2000 and January 2026. The search utilized Boolean operators: (("animal-assisted therapy" OR "animal-assisted intervention") AND ("major depressive disorder" OR "depression") AND ("cortisol" OR "oxytocin" OR "HPA axis")).

Inclusion Criteria

Inclusion criteria were as follows: (i) Peer-reviewed original research or meta-analyses; (ii) Studies involving human participants diagnosed with MDD or elevated depressive symptoms; (iii) Quantitative measurement of endocrine biomarkers (cortisol or oxytocin).

Exclusion Criteria

Exclusion criteria were: (i) Qualitative studies without biomarker data and (ii) Theses, editorials, or conference abstracts. Two independent reviewers screened titles and abstracts; conflicts were resolved through discussion. Due to high heterogeneity in intervention formats (species, duration, and assay methods), a formal meta-analysis was not feasible, and a narrative synthesis was applied.

Review of neurobiological mechanisms and biomarker evidence

Sensory Mechanisms: The Role of C-Tactile Afferents

The physiological efficacy of AAIs is deeply rooted in specific neuroanatomical pathways associated with affective touch. Stroking an animal stimulates CT afferents, specialized, low-threshold unmyelinated nerve fibers located in human hairy skin specifically tuned to slow, rhythmic tactile stimulation [13]. Unlike discriminative touch pathways that project to the somatosensory cortex for spatial localization, CT afferents project directly to the insular cortex, a region integral to interoception, emotional processing, and social bonding [13,14]. Activation of this pathway facilitates the immediate release of endogenous neurochemicals and bypasses high-level cognitive barriers, directly influencing emotional homeostasis [14,15]. For patients with MDD, this non-judgmental tactile contact provides essential sensory stimulation often absent in traditional clinical settings, offering a potent non-verbal mechanism for anxiety reduction [14,16].

The Oxytocinergic System in AAIs

The therapeutic potential of AAIs is significantly mediated by the stimulation of the oxytocinergic system. In MDD, peripheral oxytocin levels are frequently diminished, correlating with impaired social cognition and heightened anxiety [11,14]. Interaction with animals, particularly through sustained eye contact and affective touch, triggers the release of endogenous oxytocin [14-16]. This neuropeptide acts as a natural buffer, promoting emotional stabilization and prosocial behavior, which are often compromised in TRD.

Modulation of the HPA Axis: Cortisol Evidence

A primary objective of AAIs in depressive populations is the stabilization of the HPA axis. Chronic hypercortisolemia is a well-documented biomarker of MDD [7,12]. Recent studies indicate that even brief, 10-to-20-minute sessions with therapy dogs can lead to a significant rapid reduction in salivary cortisol levels [9,10,17]. This suppression suggests that AAIs serve as an acute physiological de-stressor, potentially mitigating the long-term neurotoxic effects of elevated cortisol on the hippocampus.

Species-Specific Outcomes and Interaction Durations

Evidence suggests that while canine-assisted interventions (AAT/AAA) are the most widely studied and show consistent results in cortisol reduction [6,10,18], equine-facilitated learning has also demonstrated significant impacts on basal cortisol levels [19]. The duration of interaction plays a crucial role; sessions exceeding 20 minutes typically yield more stable neuroendocrine shifts compared to sporadic, brief contacts [20,21].

A summary of the neuroendocrine effects observed in key studies is presented in Table 1.

**Table 1:** Summary of Neuroendocrine Responses to Animal-Assisted Interventions in Selected Studies. HAI: Human-animal interaction

Study (Reference)	Population & Sample Size (N)	Intervention Type & Animal Species	Primary Biomarkers / Measures Measured	Main Findings & Statistical Significance
Beetz et al. (2012) [6]	Systematic Review (69 studies)	Human-Animal Interaction (Various)	Oxytocin & Cortisol	Evidence across multiple studies suggests that HAI leads to increased oxytocin and decreased cortisol levels.
Pendry et al. (2014) [17]	Adolescents (5th–8th graders) (N=131)	EFL (Equine/Horses)	Salivary Cortisol	Significant decrease in afternoon cortisol and total daily concentration (p = 0.017) compared to control.
Scopa et al. (2019) [19]	Human-horse dyads (N=40)	EAI (Equine)	Salivary Cortisol & HRV	Significant reduction in physiological stress markers and evidence of "emotional transfer" between species.
Gee et al. (2017) [20]	Review of studies (School settings)	HAI (Primarily Canine)	Cortisol & Executive Function	Proposes the "priming effect"; HAI reduces physiological stress, facilitating improved therapeutic engagement.
Wesenberg et al. (2019) [21]	Residents with dementia (N=19)	AAT (Canine)	Social behavior & Positive emotions	Significantly longer and more frequent social interaction and positive emotions (p < 0.05) during AAT.

Discussion

Comparison With Previous Literature

The findings of this narrative review align with previous meta-analyses, such as the landmark study by Nimer and Lundahl [5], which demonstrated that AAT yields moderate-to-high effect sizes in reducing depressive symptoms. However, our review extends these findings by bridging the gap between behavioral outcomes and neurobiological mechanisms. While earlier reviews primarily focused on subjective well-being [18], our synthesis emphasizes the objective modulation of the HPA axis. The consistent reduction in cortisol levels observed across various studies [10,17,19] suggests that AAIs provide a quantifiable physiological de-stressor effect.

Clinical Implications: The "Priming Effect"

The neurobiological shifts identified, specifically the increase in oxytocin and the downregulation of the HPA axis, suggest that AAIs may serve as a "priming" strategy for traditional clinical treatments [20]. For patients with MDD, particularly those with TRD, high levels of cortisol can impair cognitive flexibility. By acutely reducing physiological arousal and increasing social openness through oxytocin release, AAIs may lower the patient's internal barriers to therapy [14,20].

## Conclusions

The neuroendocrine evidence synthesized in this review confirms that AAIs provide a measurable, biologically grounded adjunctive treatment for patients with MDD. The stabilization of the HPA axis and the upregulation of the oxytocinergic system represent key mechanisms through which human-animal interactions mitigate symptoms of stress, anxiety, and social withdrawal. Furthermore, the activation of specialized sensory pathways, such as CT afferents, underscores the importance of tactile contact in emotional regulation.

While the acute physiological benefits of AAIs are well-documented, the long-term sustainability of these effects remains a critical area for future investigation. To integrate AAIs fully into evidence-based psychiatric practice, standardized clinical protocols regarding intervention "dosage" and species-specific applications are essential. Transitioning from short-term encounters to consistent, bond-based programs may offer the most significant potential for long-term neuroplastic remodeling and improved quality of life for individuals suffering from depression.
